# The Late Dr. J. Marion Sims

**Published:** 1884-03

**Authors:** 


### THE LATE DR. J. MARION SIMS.

The announcement of the death of J. Marion Sims on the 13th of November, 1883, produced a greater shock and a more extended and perceptible gloom in the medical world, than the death of any man connected with any department of the medical profession during the present century. When we say the medical world we mean it, as Dr. Sims was as well known and as much admired for his labors and advances in the profession in every civilized country as in his wn native land.

We will not in this connection attempt a history of his labors—a detail of his startling advances and successes, as that has been done both in Europe and America by pens and tongues more eloquent than ours. Neither will we attempt an eulogy upon this great and good man, as that has also been done by hundreds and thousands in the past four months—but desire simply to say that to know him was to love him, and to be in his presence was to feel that you were in the presence of a genius and an honest man, without vanity or show, and one that could not be induced to knowingly deceive his fellow-man, and more particularly members of the medical profession. If by articles published in medical journals, monographs, orally or otherwise, he had promulgated an idea or principle, that later he found would not stand the test, he was the first to correct it, and never happy until he had accomplished it publicly.

I well recollect meeting him at his home in New York, some years ago, and found him jubilant over the idea that he had at last solved the problem, as to the best mode of drainage after an ovariotomy, with what he styled the "cock-spur drainage tube." A few months later I met him again, and he was equally eloquent in condemnation of his former pet, and was at the time preparing an article for publication, warning the profession against the "cock-spur drainage tube," as it had failed to accomplish what he believed it would. He was usually the first to detect an error in his propositions and the first to correct them. How different the majority of those who make suggestions that are new, either in medicine or surgery. In presenting their propositions they become so much enthused with the new idea that they never stop, or attempt a careful criticism of its action, and are usually the last to admit their error. Much more could be said in evidence of this noble trait of character of Dr. Sims, but enough has been said, both orally and by manuscript, within the past few months.

The time has arrived when his friends and admirers should adopt something more durable than eulogies upon his life and sketches of his noble work. Let us, as is suggested by the physicians of New York in a circular to the profession and citizens of the world, contribute our mite to the erection of a monument to his memory, which will indure for ages. Let no one feel that he or she is not called on for the reason that they are not members of the profession, but let it be understood that all who admire his great genius, his goodness of heart, and above all the noble work he accomplished, feel that their offering will be as acceptable as that coming from a member of his profession. As will be seen from the circular, Dr. H. F. Campbell, of Augusta, has been appointed to receive subscriptions in Georgia. His agent in Atlanta is Dr. D. H. Howell, and contributions, either from the city or surrounding country, will be thankfully received and properly forwarded with list of names of contributors.

### THE SIMS MEMORIAL FUND

*To the Medical Profession and Others throughout the World:*

The great achievements of Dr. J. Marion Sims call for some more lasting than obituaries and eulogies. To him medical science is indebted for much brilliant and original work, especially in gynecological surgery. Those who have been benefited by his teachings and new operations, and such as have had the direct advantage of his personal skill, are among the first to recognize and acknowledge this debt.

To him is due the honor of giving the first strong impulse to the study of gynecological surgery in America.

It is believed that the medical profession everywhere, the vast number who owe their relief from suffering directly to him, and those who realize the benefits he first made possible, will gladly unite thus to honor the man through whose original and inventive genius such blessings have been conferred upon humanity.

At the suggestion of many friends, therefore, the subjoined committee has been organized, and it is proposed that a suitable monument be erected to his memory in the city of New York.

To this end the active co-operation of the medical profession, and the many other friends of Dr. Sims throughout the world, is respectfully solicited. Contributions of one dollar and upward may be forwarded to the JOURNAL, which has been constituted the treasury of this fund.—*The Medical Record, New York.*

FORDYCE BARKER, M.D., *Chairman.*
GEORGE F. SHRADY, M.D., *Secretary.*

Thomas Addis Emmet, M.D., New York.

T. Gaillard Thomas, M.D., New York.

William T. Lusk, M D., New York.

William M. Polk, M.D., New York.

Paul F. Mundi, M.D., New York.

S. O. Vander Poel, M.D., New York.

Frank P. Foster, M.D., New York.

Samuel D. Gross, M.D., Philadelphia, Pa.

William Goodell, M.D., Philadelphia, Pa.

James R. Chadwick, M.D., Boston, Mass.

William H. Byford, M.D., Chicago, Ill.

A. Reeves Jackson, M.D., Chicago, Ill.

Thad. A. Reamy, M.D., Cincinnati, O.

C. D. Palmer, M.D , Cincinnati, O.

George J. Engelmann, M.D., St. Louis, Mo.

R. Beverly Cole, M.D., San Francisco, Cal.

H. F. Campbell, M.D., Augusta, Ga.

R. B. Maury, M.D., Memphis, Tenn

E. S. Lewis, M.D., New Orleans, La.

J. T. Searcy, M.D., Tuskaloosa, Ala.

R A. Kinloch, M D., Charleston, S. C.

Hunter McGuire, M.D., Richmond, Va.

S. C. Busey, M.D., Washington, D. C.

Harvey L. Byrd, M.D., Baltimore, Md.

W. J. Howard, M.D., Baltimore, Md.

